# Agreement Analysis Among Hip and Knee Periprosthetic Joint Infections Classifications

**DOI:** 10.3390/diagnostics15091172

**Published:** 2025-05-04

**Authors:** Caterina Rocchi, Marco Di Maio, Alberto Bulgarelli, Katia Chiappetta, Francesco La Camera, Guido Grappiolo, Mattia Loppini

**Affiliations:** 1 Department of Biomedical Sciences, Humanitas University, Pieve Emanuele, 20090 Milan, Italy; caterina.rocchi@st.hunimed.eu (C.R.); alberto.bulgarelli@humanitas.it (A.B.); katia.chiappetta@humanitas.it (K.C.); 2 Dipartimento di Scienze Mediche, Chirurgiche e della Salute, Università degli Studi di Trieste, Piazzale Europa, 1, 34127 Trieste, Italy; dimaiomarco95@gmail.com; 3 IRCCS Humanitas Research Hospital, Rozzano, 20089 Milan, Italy; francesco.lacamera@humanitas.it (F.L.C.); guido.grappiolo@mac.com (G.G.)

**Keywords:** PJI, diagnosis, knee, hip, biomarkers

## Abstract

**Background/Objectives**: A missed periprosthetic joint infection (PJI) diagnosis can lead to implant failure. However, to date, no gold standard for PJI diagnosis exists, although several classification scores have been developed in the past years. The primary objective of the study was the evaluation of inter-rater reliability between five PJI classification systems when defining a patient who is infected. Two secondary outcomes were further examined: the inter-rater reliability assessed by comparing the classifications in pairs, and the evaluation of each classification system within the subcategories defined by the World Association against Infection in Orthopaedics and Trauma (WAIOT) definition. **Methods**: Retrospectively collected data on patients with knee and hip PJIs were used to assess the agreement among five PJI scoring systems: the Musculoskeletal Infection Society (MSIS) 2013 definition, the Infection Consensus Group (ICG) 2018 definition, the European Bones and Joints Infection Society (EBJIS) 2018 definition, the WAIOT definition, and the EBJIS 2021 definition. **Results**: In total, 203 patients with PJI were included in the study, and the agreement among the examined scores was 0.90 (Krippendorff’s alpha = 0.81; *p*-value < 0.001), with the MSIS 2013 and ICG 2018 classification systems showing the highest agreement (Cohen’s Kappa = 0.91; *p*-value < 0.001). **Conclusions**: There is a strong agreement between the major PJI classification systems. However, a subset of patients (*n* = 11, 5.42%) still falls into a diagnostic grey zone, especially in cases of low-grade infections. This highlights the need for enhanced diagnostic criteria that incorporate tools that are available even with limited resources, and the potential of artificial intelligence-based techniques in improving early detection and management of PJIs.

## 1. Introduction

Periprosthetic joint infection (PJI) is one of the most significant complications following total joint arthroplasty and still represents a public health issue, representing a challenge both in terms of diagnosis and treatment [1]. Underdiagnosing PJI leads to inadequate treatment, with a high risk of severe consequences; conversely, its overdiagnosis can expose patients to unnecessary therapeutic measures, namely long antibiotic regimens and complex surgeries [2]. The diagnosis of PJI is based on a combination of clinical findings, laboratory results from the peripheral blood and synovial fluid, microbiological cultures, the histological evaluation of periprosthetic tissue, and intraoperative findings.

Different classification systems have been developed in the past decade to standardize the definition of PJI using a combination of criteria. The landmark definition of periprosthetic joint infections was set in 2011, introducing the Musculoskeletal Infection Society (MSIS) classification as the first widely adopted standard that revolutionized how PJIs were diagnosed and managed [3]. The classification not only established the initial benchmark for identifying infections but also set the thresholds that have been incorporated into almost all the subsequent definitions, underscoring its lasting impact on the field. In 2013, the International Consensus Meeting further developed the MSIS definition and introduced the concept of major and minor criteria, which are fundamental in distinguishing between evident infections with a sinus tract and subtle ones. The new definition also provided a clear definition of the presentation of subtle infections, where the diagnosis is purely clinical, by analyzing the inflammatory markers in both synovial fluid and blood serum and further refining the thresholds for the minor criteria [4]. The consensus group overseeing this modification noted that a possible limitation of the classification was represented by PJIs caused by less virulent organisms, usually providing negative microbiological tests and only subtle clinical hints. After 5 years, the emergence of new diagnostic tests and new techniques led to a new definition by the Infection Consensus Group, who kept the major criteria of the MSIS, modified the weight of each minor criteria, and added new tests as D-dimer, prosthesis sonication, and alpha-defensin [5]. In 2019, the World Association against Infection in Orthopaedics and Trauma (WAIOT) [6] proposed an entirely new approach. They classified all the tests for PJI into “rule-in” or “rule-out” tests based on their specificity and sensitivity. The results of these tests, combined with the initial clinical presentation, could lead to four possible outcomes: “aseptic”, “biofilm-related”, “low-grade”, and “high-grade” PJI. The acknowledgement of the existence of “low-grade” and “biofilm-related” infections, characterized by the absence of acute inflammatory signs and a more subtle presentation, led to a new approach in PJI diagnosis and management.

In 2018, the European Bone and Joint Infection Society (EBJIS) developed a new classification system for PJIs that incorporated the use of 16S rRNA gene sequencing, which can detect a wider range of pathogens, particularly in culture-negative PJIs [7]. Lastly, in 2021, the EBJIS developed a new classification system for PJIs, classifying cases as “infection unlikely”, “infection likely”, and “infection confirmed”. This new classification system employed molecular markers, such as alpha-defensin, and introduced nuclear imaging as both a rule-in (positive scintigraphy) and rule-out (negative three-phase isotope bone scan) test [8].

Despite extensive efforts by the scientific community to develop new classification systems, a definitive reference standard remains elusive. One significant challenge is presented by borderline cases, particularly those with culture-negative infections, which tend to exhibit a more insidious clinical presentation. Notably, these cases are typically associated with lower mean synovial and serum white blood cell (WBC) counts, C-reactive protein (CRP) levels, and erythrocyte sedimentation rates (ESRs) when compared with culture-positive cases [9]. In addition, culture-negative infections are often caused by atypical pathogens forming biofilms such as fungi (46%), mycobacteria (43%), and atypical bacteria (21%) [10]. This aspect is not of secondary importance, since in the literature, a 42% rate of culture-negative PJI failing the therapeutic regimen has been reported, and 53% of these eventually became culture-positive [11]. Bacterial genome sequencing would make the diagnosis of infections easier and more accurate [2,12,13]; unfortunately, an extensive use of this technique is not cost-effective at the moment [14,15]. Given these circumstances, PCR-based tools could play a fundamental role in identifying the pathogens causing culture-negative infections. Indeed, techniques such as 16S rRNA PCR, quantitative PCR, and multiplex PCR allow the detection of the bacterial genome, even in the absence of culturable organisms, with increased diagnostic sensitivity.

Additionally, artificial intelligence (AI)-based approaches hold the potential to improve the early prediction of complications and implant failure. Combining deep learning and machine learning has shown promising results in detecting the failure of hip prosthesis from plain radiographs, with a very high degree of precision [16].

The aim of the study was to investigate the agreement among the five major classification systems used to define both hip and knee PJIs. The primary objective of the study was the evaluation of inter-rater reliability for all the classification systems when defining an patient who is infected. Two secondary outcomes were further examined: the inter-rater reliability assessed by comparing classifications in pairs, and the evaluation of each classification system within the subcategories defined by the WAIOT classification, with the goal of identifying potential significant disagreement between these systems.

## 2. Materials and Methods

### 2.1. Study Design

A retrospective observational study was conducted based on data from all the consecutive cases of PJI of the hip and knee at our tertiary orthopedic referral center between October 2016 and December 2019.

The target population comprised eligible patients for revision surgery for total hip arthroplasty (THA), total knee arthroplasty (TKA), or partial knee arthroplasty (PKA). The inclusion criteria required that patients experienced pain for at least 3 months and had undergone a minimum set of microbiological analyses, including synovial fluid cultures, intraoperative sampling, and prosthesis sonication, to ensure sufficient clinical and laboratory data for determining the presence or absence of a PJI according to all the classification systems (MSIS 2013, ICG 2018 definition, EBJIS 2018 definition, WAIOT, and EBJIS 2021). The exclusion criteria were the use of antibiotics, glucocorticoids, and anti-histaminic therapy within 2 weeks prior to surgery; rheumatoid arthritis and other rheumatic disorders; and revision surgery for spacer removal and reimplantation, metallosis, prosthetic dislocation, periprosthetic fracture, limb length discrepancy, prosthetic rupture, and the wear of polyethylene.

Data from the included patients were then used to define the presence or absence of PJI in each case, employing all five classification systems (MSIS 2013 [4], WAIOT [6], ICG 2018 definition [5], EBJIS 2018 [7], EBJIS 2021 [8]).

Following this initial categorization, the results were further stratified using the WAIOT classification into “aseptic”, “biofilm-related”, “low-grade”, and “high-grade” infections, with contaminated samples included within the “aseptic” category. For the EBJIS 2021 classification, an initial dichotomous classification was performed by grouping the “likely” and “confirmed” cases together as “infected.” Subsequently, the “likely” and “confirmed” cases were analyzed as separate categories when comparing the classifications in pairs. This methodology enabled a more precise analysis, without oversimplification.

The enrolled patients were managed by our center with the “one-stage” or “two-stage” revision, according to the diagnosis of PJI formulated using the EBJIS 2018 criteria [7]. Therefore, the results of the analysis did not affect the diagnosis of a PJI and consequently the choice of the type of treatment for each patient.

### 2.2. Laboratory Methods

Blood samples and synovial fluid were collected pre-operatively and intra-operatively, respectively, for THA, TKA, and PKA revision surgery from all patients. CRP, ESR, and D-dimer were measured following the standard procedures in use in the Institutional Clinical Laboratory. Multiple biopsies were performed intraoperatively to increase the sensitivity of the cultural methods and to distinguish contaminating microorganisms from pathogens. Each biopsy of periprosthetic tissue was collected with separated instrumentation and stored in a dedicated container, in order to minimize the risk of cross-contamination of the samples sent for the culture examination. To obtain a comprehensive evaluation of the infective status of the patient, three sets of cultures were obtained for each patient’s periprosthetic tissue samples, synovial fluid, and prosthesis sonicate. In addition to the classical culture, each sample underwent a culture for anaerobic bacteria for 14 days and a second one for filamentous fungi. The prosthetic devices were subjected to mechanical forces to detach the biofilm: the prosthesis was immersed in Ringer’s solution and subjected to ultrasound waves at 50 Hz for 5 min, then the resulting fluid was sent immediately to the microbiology laboratory for further analysis. A leukocyte count on the synovial fluid was performed using manual microscopy, within 30 min from the arrival of the sample in the laboratory. Before analysis, the samples were gently mixed to reverse possible sedimentation. A sample with fewer than 500 cells/μL was considered to be negative.

### 2.3. Statistical Analysis

The statistical analysis was performed using STATA version 17, with the SSC kappaetc package. The graphical representation was developed using Graphpad PRISM version 10. For each reported classification, each patient was classified according to binary variables: 1 for “infected”, following the classification criteria, and 0 for “not infected”, based on the same criteria; this process generated five columns according to the MSIS 2013, WAIOT, ICG 2018, EBJIS 2018, and EBJIS 2021 (“likely” + “confirmed”) classification systems. To enhance the methodological consistency, the analysis was performed by considering EBJIS as three separate categories when comparing the raters in pairs: “EBJIS 2021 binomial” (considering both “likely” and “confirmed” cases as infected), EBJIS 2021 “likely”, and EBJIS 2021 “confirmed” cases. This approach allowed us to achieve a complete agreement analysis without forcing the EBJIS 2021 system to fit into a dichotomous framework, allowing us to appreciate the statistical discrepancies in the paired analysis between the different categories proposed by the EBJIS classification system.

When three or more raters were evaluated together for inter-rater reliability, the statistical test of choice was the Krippendorff’s alpha [17,18], which is more flexible and accounts for missing data. When only two raters were evaluated, Cohen’s Kappa and Gwet’s AC1 statistic were employed [19]. It must be noted that when one category is much more prevalent than another, Cohen’s Kappa may yield low values. In contrast, Gwet’s AC1 adjusts the imbalanced agreement values, leading to a higher agreement. The interpretation of these values was left to standard medical knowledge, reported by Landis and Koch [20]. This scale has been criticized in non-medical fields, where a stricter criterion of agreement is often required (over 0.8 to establish agreement), but for the purpose of the study, this consolidated approach was considered acceptable. The statistical difference between groups was assessed using the Friedman test for the multiple-group comparisons. The McNemar’s Exact test was used for the two-group comparisons.

## 3. Results

### 3.1. Demographics

From October 2016 to December 2019, a total of 203 patients referred to our institute were enrolled in the study; after the application of the exclusion criteria, the final sample consisted of 203 patients, including both THA (*n* = 159) and TKA or PKA (*n* = 44) revisions. Within the sample, 59 patients had a PJI according to the MSIS criteria, 65 according to the ICG 2018 classification, 89 according to the EBJIS 2018 definition, 72 following the WAIOT criteria, and 73 according to the EBJIS 2021 classification. The subdivision of patients following the WAIOT indications resulted in 125 “aseptic patients”, 57 “biofilm-related infections”, 5 “low-grade infections”, and 10 “high-grade infections”. Additionally, 6 patients could not clearly be classified according to the WAIOT definition. Patients’ demographics were reported in Table 1.

### 3.2. Agreement Among Classifications

**Figure 1:**
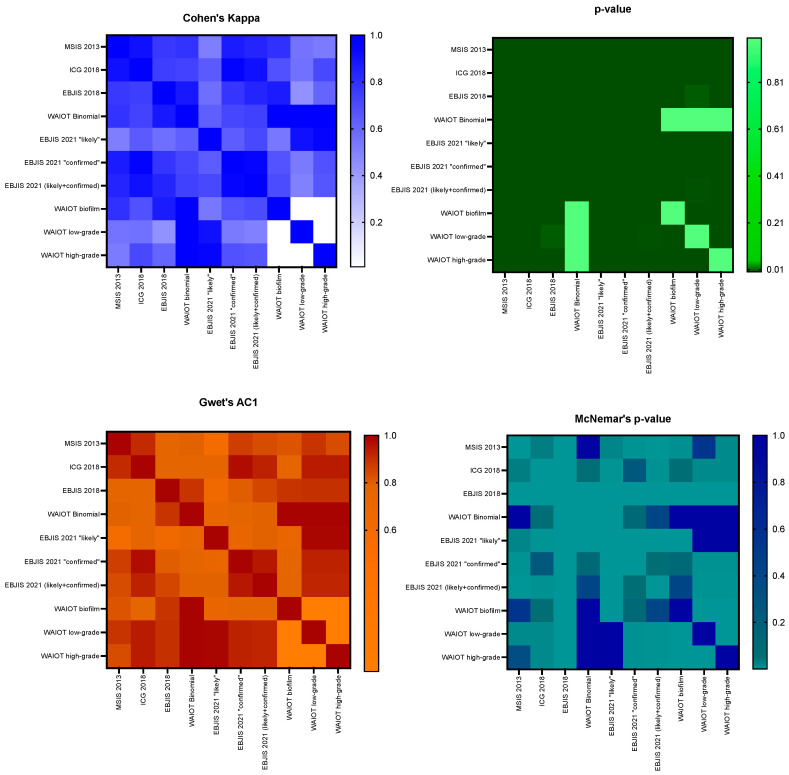
Agreement analysis between the different classification systems, with the corresponding *p*-values and Mc Nemar’s *p*-values compared in the heatmaps above. *p*-values and McNemar’s *p*-values < 0.05 were considered statistically significant.

The classification systems examined agreed in most cases, resulting in an elevated agreement rate (0.90); if the value was corrected by the Krippendorff’s Alpha, the result was 0.81, still showing a substantial agreement, with a *p*-value of <0.001. The analysis of the paired classifications is reported in Table 2 and Figure 1, with the MSIS 2013 and ICG 2018 pair showing the highest agreement.

**Figure 2:**
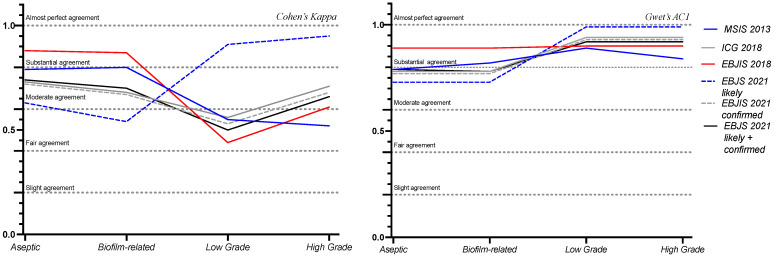
Comparison between the major PJI classification systems (MSIS 2013, ICG 2018, EBJIS 2018, EBJIS 2021), represented by the lines, and their agreement with the WAIOT categories, displayed in the x axis.

All the classification systems were further analyzed to observe discrepancies among the categories coded by the WAIOT classification, as can be seen in Table 3 and Figure 2.

### 3.3. Clinical Implications of Agreement Analysis

Between the examined classification system pairs, there were some cases in which, despite an agreement coefficient above 0.90, the McNemar’s *p*-value still indicated significant discrepancies (MSIS2013 and ICG2018, ICG2018 and EBJIS 2021). The MSIS 2013 and ICG 2018 classification systems displayed a very high agreement (0.91) with a significant McNemar’s *p*-value (0.031). A total of six patients were classified as infected according to ICG 2018 but aseptic according to MSIS 2013. Among them, three were managed as septic and three as aseptic by our structure based on the EBJIS 2018 criteria. None experienced implant failure or required revision procedures within five years. Conversely, five patients were categorized as infected according to EBJIS 2021 but not according to ICG 2018 (Cohen’s kappa = 0.91, McNemar’s *p*-value = 0.08). Three received a two-stage implant revision, while two were managed as aseptic. One of these patients, managed as aseptic by our structure, underwent an implant revision for septic loosening in 2021.

## 4. Discussion

The analysis of agreement between the PJI classification systems revealed “high” and “moderate” levels of agreement across the different criteria. Specifically, the MSIS 2013 and ICG 2018 classification systems showed an almost perfect agreement, with both Cohen’s Kappa and Gwet’s AC1 equal to 0.91 (*p*-value < 0.001; McNemar’s *p*-value = 0.031), indicating a near-complete overlap in their rating systems. This suggests that these systems effectively classify infections in a consistent manner. In contrast, several other comparisons fell within the “moderate” to “substantial” agreement range, notably the MSIS 2013 vs. EBJS 2021, yielding a Cohen’s Kappa of 0.51 (*p*-value < 0.001), likely due to the differing thresholds in the synovial fluid analysis.

In some cases, despite a substantial agreement indicated by the Cohen’s Kappa and Gwet’s AC1 tests, the presence of McNemar’s *p*-values < 0.05 indicated statistically significant discrepancies between the classification systems, even in cases of high agreement coefficients. These discrepancies mean that even in cases of a high agreement between the two raters, there was still a proportion of borderline cases (McNemar’s *p*-value < 0.05), which are difficult to identify and manage in clinical practice. Indeed, MSIS 2013 vs. ICG 2018 showed a high agreement (Cohen’s Kappa = 0.91), but they still diverged in a small subset of patients, which represented clinically important borderline cases. Indeed, six patients were classified as “infected” according to the ICG 2018 but not according to the MSIS 2013 classification system. This difference likely depended on differences in the thresholds indicated by the two classification systems. Out of the six cases, three were managed as aseptic and three as infected by our structure, in line with the EBJIS 2018 definition. All these cases displayed good clinical outcomes in the way that they were classified and managed by our structure, according to the EBJIS 2018 classification system. Two of these patients died approximately three years after the operation due to causes unrelated to the implant, with intact prosthetic devices at the moment of death. Notably, within the “aseptic” group, a 70-year-old male was managed as aseptic and underwent revision of the acetabular component and ceramic head. The patient had positive sonication fluid for Propionibacterium Acnes. Despite this, he had no sequelae. This case exemplifies a situation in which classifying and managing the patient as septic would have exposed him to overtreatment. Indeed, distinguishing between mere contaminants and a clinically significant biofilm represents a challenge [21].

Additionally, five patients were categorized as infected according to EBJIS 2021 but not according to ICG 2018 (Cohen’s kappa = 0.91, McNemar’s *p*-value = 0.08). Three of them received a two-stage implant revision, while two were managed as aseptic. One of these patients, a 62-year-old male, underwent implant re-revision for septic loosening three years after his revision surgery. At the time of the first revision, our institution managed the case as aseptic. However, according to the EBJIS 2021 classification, the patient fell into the “infection likely” category. Despite negative cultures, his synovial WBC count was 2.400 and he presented with mild elevations in CRP and ESR levels, which were equal to 3.07 mg/L and 40 mm/hr, respectively. With the absence of positive cultures and nonspecific elevations in WBCs and inflammatory markers, this patient constituted an excellent example of borderline case, identified as “likely infected” according to the EBJIS 2021 classification system but aseptic according to ICG2018. Three years later, the patient developed septic loosening, requiring a re-revision surgery. Intraoperative cultures confirmed a chronic PJI, with 9/9 intraoperative tissue samples and the prosthesis sonication fluid testing positive for methicillin-sensitive Staphylococcus epidermidis. This case highlights the challenges of borderline cases, which may initially not meet the universal criteria for a PJI diagnosis and present with negative cultures but can still progress to an overt infection and implant failure [22,23].

Some systems, like EBJIS 2018, showed frequent divergences, as evidenced by McNemar’s *p*-values of less than 0.001. These discrepancies arise from the unique criteria of this classification system, reflecting the role of employing NGS techniques for diagnosing low-grade infections [24]. Indeed, the recognition of very low-grade infections is one of the main issues related to PJI diagnosis in the clinical practice. This diagnostic difficulty has been investigated by Moojen, D.J.F., who described a rate of 4% to 13% of PJI from low-virulence bacteria (Coagulase-Negative Staphylococci and Proprionibacterium acnes) in patients with a preoperative diagnosis of aseptic loosening [25].

In our study, the EBJIS 2021 displayed the highest capability of diagnosing WAIOT “low-grade” infections, which fell under the “infection likely” subgroup (Cohen’s kappa = 0.91, *p*-value < 0.001, Gwet’s AC1 = 0.99, McNemar’s *p*-value = 0.004). Indeed, the EBJIS 2021 has introduced a novel approach to diagnosing PJIs, aiming to enhance the detection of low-grade infections. A study by Sousa et al. showed that when compared with other PJI definitions, the EBJIS 2021 showed the highest agreement between pre-operative and definitive classification (k = 0.9, CI 0.8–0.9) and was better at ruling out PJIs with an infection-unlikely result (sensitivity 89%, negative predictive value 90%) [26]. In a comparative analysis, Sigmund et al. assessed the EBJIS 2021 criteria against other PJI definitions. The EBJIS definition demonstrated the highest agreement between preoperative assessments and definitive classifications, with a kappa value of 0.9 (95% CI 0.8–0.9) [27]. These findings suggest that the EBJIS 2021 criteria are particularly effective in identifying low-grade PJIs, offering a more sensitive diagnostic tool compared with the previous definitions.

Notably, the MSIS 2013 and ICG 2018 demonstrate a high agreement in this dataset; an explanation for this could be that they share similar criteria developed by the same research group. Additionally, the EBJIS 2021 likely vs. EBJIS 2021 confirmed criteria exhibited a substantial agreement, but still showed statistically significant classification differences (Cohen’s Kappa = 0.64, *p*-value < 0.001, Gwet’s AC1 = 0.75, McNemar’s *p*-value < 0.001); these differences could be attributed to the different thresholds used to define infections.

The EBJIS 2021 “likely” category apparently has limited success in recognizing the WAIOT’s biofilm-related infections (Cohen’s Kappa = 0.54, *p*-value < 0.01). Despite this, it must be noted that the Gwet’s AC1 test demonstrates substantial levels of agreement (Gwet’s AC1 = 0.73, McNemar’s *p*-value < 0.01). Thereby, the apparent discrepancy between the two agreement tests could be explained by the different prevalences of the two groups, which are better accounted for using the Gwet’s AC1 test. Furthermore, the fact that the EBJIS 2021 confirmed category displays a substantial agreement with the biofilm-related infections (Cohen’s Kappa = 0.67, *p*-value < 0.01, Gwet’s AC1 = 0.77, McNemar’s *p*-value = 0.11) may lead to the thinking that biofilm-related infections are rather included in the confirmed group, with non-invasive methods not always being effective in detecting biofilm until it reaches a more active state. Indeed, the best outcomes in identifying biofilm-related infections are yielded by the EBJIS 2018 criteria (Cohen’s Kappa = 0.87, Gwet’s AC1 = 0.89, *p*-value and McNemar’s *p*-value < 0.001). This is probably due to the incorporation in the classification system of novel techniques, including next-generation sequencing (NGS). Biofilm-related infections pose a significant challenge, requiring advanced and expensive diagnostic techniques while carrying the constant risk of overdiagnosis. Given these challenges, it appears unlikely that an effective screening method for biofilm infections can be established with current methodologies, at least not until these infections transition to a more active phase.

When infections are left untreated or are not managed correctly, biofilms can persist, leading to a cascade of severe outcomes. These persistent infections often result in prolonged pain and irreversible complications, such as the loosening of prosthetic devices, limb amputations, and even death. Beyond the direct impact on patient health, these complications impose substantial financial and social burdens on healthcare systems and society as a whole [28,29]. The disagreement concerning biofilm detection between traditional PJI classification systems highlights the need for innovative diagnostic approaches. AI could play a crucial role in the early diagnosis of PJIs, identifying patient-related risk factors (with the main predictors being total length of stay > 3 days, patient weight > 94.3 kg, ASA score 4 or higher, preoperative platelet count < 249,890/mm^3^, and preoperative sodium levels < 139.5 mEq/L) [30]. A machine-learning model combining 21 features, including imaging indicators (X-ray and CT), has shown a higher sensitivity and accuracy than the ICG 2018 both in the development (90.6% vs. 76.1%, *p* = 0.032; 94.5% vs. 86.7%, *p* = 0.020) and in the internal validation cohort (84.2% vs. 78.6%; 94.6% vs. 81.8%) [31]. Additionally, an AI-based framework has been developed to diagnose PJIs based on 99mTc-MDP dynamic bone scintigraphy, with high diagnostic performances (AUC of 0.96 for periprosthetic knee infections and 0.91 for hip infections) [29]. Integrating AI into routine clinical practice for at-risk patient populations holds significant promise. By enhancing the detection of biofilm-related PJIs, AI can lead to earlier and more accurate diagnoses. Furthermore, this technology can improve the classification of borderline cases, thereby enabling clinicians to customize treatment strategies more effectively. The result is not only better patient outcomes, but also a more efficient use of healthcare resources.

The major strengths of this study are its large sample size and the fact that several classification systems were included in the analysis. However, the main limitations are the retrospective collection of data and the absence of an adequate gold standard for PJI diagnosis. This means that, while agreement comparisons between the different classification systems are possible, there is no univocal definition of truly infected and non-infected cases to compare the results with.

## 5. Conclusions

The major PJI classification systems showed a strong agreement. Despite this, even among systems that otherwise show a high agreement, such as the MSIS 2013 and ICG 2018, a subset of patients falls into a diagnostic grey zone where differences in thresholds can lead to divergent classifications. Diagnosing these borderline cases, often involving low-grade infections, poses a critical challenge, with the persistent risk of both overdiagnosis and underdiagnosis. The difficulty in diagnosing unclear clinical scenarios highlights the need for enhanced diagnostic criteria, which are able to incorporate all the potential tools available and allow diagnosis even with limited data and resources, since not all microbiological and molecular imaging-based diagnostic tools are available in every hospital. The potential integration of AI-based techniques to refine the early detection and management of PJIs in at-risk patient categories seems quite promising.

## Figures and Tables

**Table 1:** Patients’ demographic data. M: male sex; BMI: body mass index; ASA: American Society of Anesthesiology score; CCI: Charlson Comorbidity Index; IQR: interquartile range.

Demographic Characteristics	Value (IQR)
Sex (M)	93 (45.81%)
BMI	27.11 (16.65–41.02)
Age at operation	66.93 (18.32–90.31)
Charlson Comorbidity Index	
High Comorbidity Profile (CCI > 2)	116 (57.14%)
Low Comorbidity Profile (CCI ≤ 2)	87 (42.86%)
ASA score	
ASA score I	30 (14.78%)
ASA score II	121 (59.61%)
ASA score III	52 (25.62%)

**Table 2:** Agreement between classification pairs.

Compared Classifications	Cohen’s Kappa	*p*-Value	Gwet’s AC1	McNemar’s *p*-Value	Interpretation
MSIS 2013—ICG 2018	0.91	<0.001	0.91	0.031	Almost perfect agreement
MSIS 2013—EBJIS 2018	0.77	<0.001	0.77	<0.001	Substantial agreement
MSIS 2013—WAIOT	0.79	<0.001	0.79	1.000	Substantial agreement
MSIS 2013—EBJIS 2021	0.84	<0.001	0.84	<0.001	Almost perfect agreement
MSIS 2013—EBJIS 2021 likely	0.51	<0.001	0.57	0.022	Moderate agreement
MSIS 2013—EBJIS 2021 confirmed	0.87	<0.001	0.87	0.004	Almost perfect agreement
ICG 2018—EBJIS 2018	0.75	<0.001	0.78	<0.001	Substantial agreement
ICG 2018—WAIOT	0.73	<0.001	0.78	0.064	Substantial agreement
ICG 2018—EBJIS 2021	0.91	<0.001	0.93	0.008	Almost perfect agreement
ICG 2018—EBJIS 2021 likely	0.65	<0.001	0.76	0.003	Substantial agreement
ICG 2018—EBJIS 2021 confirmed	0.97	<0.001	0.97	0.250	Almost perfect agreement
EBJIS 2018—WAIOT	0.88	<0.001	0.89	<0.001	Almost perfect agreement
EBJIS 2018—EBJIS 2021	0.84	<0.001	0.85	<0.001	Almost perfect agreement
EBJIS 2018—EBJIS 2021 likely	0.57	<0.001	0.63	<0.001	Moderate agreement
EBJIS 2018—EBJIS 2021 confirmed	0.78	<0.001	0.8	<0.001	Substantial agreement
WAIOT—EBJIS 2021	0.74	<0.001	0.79	0.405	Substantial agreement
WAIOT—EBJIS 2021 likely	0.63	<0.001	0.73	<0.001	Substantial agreement
WAIOT—EBJIS 2021 confirmed	0.72	<0.001	0.77	0.110	Substantial agreement

**Table 3:** Agreement between the different classification systems and WAIOT categories.

Compared Classifications	Cohen’s Kappa	*p*-Value	Gwet’s AC1	McNemar’s *p*-Value	Interpretation
WAIOT BIOFILM—MSIS 2013	0.8	<0.001	0.82	0.549	Almost perfect agreement
WAIOT LOW GRADE—MSIS 2013	0.55	<0.001	0.89	0.016	Moderate agreement
WAIOT HIGH-GRADE—MSIS 2013	0.52	1.000	0.84	0.344	Moderate agreement
WAIOT ASEPTIC—MSIS 2013	0.79	<0.001	0.79	1.000	Substantial agreement
WAIOT BIOFILM—ICG 2018	0.68	<0.001	0.78	0.064	Substantial agreement
WAIOT LOW-GRADE—ICG 2018	0.56	<0.001	0.94	0.016	Moderate agreement
WAIOT HIGH-GRADE—ICG 2018	0.71	<0.001	0.94	0.016	Substantial agreement
WAIOT ASEPTIC—ICG 2018	0.73	<0.001	0.78	<0.001	Substantial agreement
WAIOT BIOFILM—EBJIS 2018	0.87	<0.001	0.89	<0.001	Almost perfect agreement
WAIOT LOW-GRADE—EBJIS 2018	0.44	0.006	0.9	<0.001	Moderate agreement
WAIOT HIGH-GRADE—EBJIS 2018	0.61	<0.001	0.9	<0.001	Substantial agreement
WAIOT ASEPTIC—EBJIS 2018	0.88	<0.001	0.89	<0.001	Almost perfect agreement
WAIOT BIOFILM—EBJIS 2021	0.7	<0.001	0.78	<0.001	Substantial agreement
WAIOT LOW-GRADE—EBJIS 2021	0.5	0.002	0.92	<0.001	Moderate agreement
WAIOT HIGH-GRADE—EBJIS 2021	0.66	<0.001	0.92	1.000	Substantial agreement
WAIOT ASEPTIC—EBJIS 2021	0.74	<0.001	0.79	<0.001	Substantial agreement
WAIOT BIOFILM—EBJIS 2021 likely	0.54	<0.001	0.73	0.022	Moderate agreement
WAIOT LOW-GRADE—EBJIS 2021 likely	0.91	<0.001	0.99	0.004	Almost perfect agreement
WAIOT HIGH-GRADE—EBJIS 2021 likely	0.95	<0.001	0.99	0.405	Almost perfect agreement
WAIOT ASEPTIC—EBJIS 2021 likely	0.63	<0.001	0.73	<0.001	Substantial agreement
WAIOT BIOFILM—EBJIS 2021 confirmed	0.67	<0.001	0.77	0.110	Substantial agreement
WAIOT LOW-GRADE—EBJIS 2021 confirmed	0.53	0.001	0.93	0.008	Moderate agreement
WAIOT HIGH-GRADE—EBJIS 2021 confirmed	0.68	<0.001	0.93	0.008	Substantial agreement
WAIOT ASEPTIC—EBJS 2021 confirmed	0.72	<0.001	0.77	0.110	Substantial agreement

## Data Availability

The data supporting the reported results can be found in a repository (Zenodo).
